# Clinical Improvement by Switching to an Integrase Strand Transfer Inhibitor in Hemophiliac Patients with HIV: The Japan Cohort Study of HIV Patients Infected through Blood Products

**DOI:** 10.2174/1874613601711010018

**Published:** 2017-04-26

**Authors:** Miyuki Kawado, Shuji Hashimoto, Shin-ichi Oka, Katsuyuki Fukutake, Satoshi Higasa, Hiroshi Yatsuhashi, Miwa Ogane, Manabu Okamoto, Takuma Shirasaka

**Affiliations:** 1 Department of Hygiene, Fujita Health University School of Medicine, Toyoake, Japan; 2 AIDS Clinical Center, National Center for Global Health and Medicine, Tokyo, Japan; 3 Department of Laboratory Medicine, Tokyo Medical University Hospital, Tokyo, Japan; 4 Division of Hematology, Department of Internal Medicine, Hyogo College of Medicine, Nishinomiya, Japan; 5 Clinical Research Center, National Hospital Organization Nagasaki Medical Center, Nagasaki, Japan; 6 AIDS Medical Center, National Hospital Organization Osaka National Hospital, Osaka, Japan

**Keywords:** HIV, AIDS, Antiretroviral therapy, HIV RNA, CD4 cell count, Integrase strand transfer inhibitor

## Abstract

**Objective:**

This study aimed to determine improvement in HIV RNA levels and the CD4 cell count by switching to an antiretroviral regimen with an integrase strand transfer inhibitor (INSTI) in patients with HIV.

**Method:**

This study was conducted on Japanese patients with HIV who were infected by blood products in the 1980s. Data were collected between 2007 and 2014. Data of 564 male hemophiliac patients with HIV from the Japan Cohort Study of HIV Patients Infected through Blood Products were available. Changes in antiretroviral regimen use, HIV RNA levels, and the CD4 cell count between 2007 and 2014 were examined.

**Results:**

From 2007 to 2014, the proportion of use of a regimen with an INSTI increased from 0.0% to 41.0%. For patients with HIV who used a regimen, including an INSTI, the proportion of HIV RNA levels <50 copies/mL significantly increased from 58.3% in 2007 to 90.6% in 2014. Additionally, the median CD4 cell count significantly increased from 380/μL to 438/μL.

**Conclusion:**

There is a large effect of switching to an antiretroviral regimen with an INSTI for Japanese patients with HIV who are infected by blood products. This suggests that performing this switch in clinical practice will lead to favorable effects.

## INTRODUCTION

Antiretroviral therapy for the treatment of human immunodeficiency virus (HIV) infection has improved and reduced HIV-associated morbidity and mortality [1-3]. Recently, integrase strand transfer inhibitors (INSTIs), as a new class of drugs, were developed [4]. In Japan, the first INSTI called raltegravir was approved for treatment of HIV infection in 2008 [5]. Several studies have reported that an antiretroviral regimen with INSTI has a high efficacy for maintaining viral suppression, and low short-term and long-term toxicities [6-10]. The guidelines for use of antiretroviral therapy in patients with HIV in the United States, Europe, and Japan include recommendations on switching to an antiretroviral regimen with an INSTI in the setting of viral suppression and viral failure [3, 5, 11]. However, the effect of this switching in clinical practice for patients with HIV on long-term antiretroviral therapy has not been sufficiently examined [3, 12].

Cohort studies are essential for providing information on clinical practices and actual long-term effects of antiretroviral treatments for patients with HIV [12, 13]. The Japan Cohort Study of HIV Patients Infected through Blood Products is one of these cohorts [14-16]. The participants in this cohort included many people with HIV infection by blood products in the 1980s. Most registrations were in the 1990s and the participants’ follow-ups are almost complete.

This study aimed to determine the effect of switching to an antiretroviral regimen with an INSTI on HIV RNA levels and the cluster of differentiation four (CD4) cell count from 2007 to 2014. We used data from the Japan Cohort Study of HIV Patients Infected through Blood Products.

## METHODS

### The Japan Cohort Study of HIV Patients Infected Through Blood Products

In Japan, a research program for people with HIV infection by use of contaminated blood coagulation factor products has been carried out since the 1993 fiscal year with the support of the Ministry of Health, Labour and Welfare [14, 15]. This program is intended to help prevent these people from developing HIV-infected symptoms in daily living by providing health management expenses. For this program, participants are requested to submit reports filled out by their treatment physician on a quarterly basis. These reports include HIV RNA levels, CD4 cell count, and administered antiretroviral drugs. If participants are diagnosed with acquired immunodeficiency syndrome (AIDS), they are excluded from this program. The Japan Cohort Study of HIV Patients Infected through Blood Products, which was based on this program, was started at the same time. Details of the cohort study are described elsewhere [14-16].

### Data Analysis

We selected a subset cohort from the Japan Cohort Study of HIV Patients Infected through Blood Products [16]. The cohort consisted of 564 male hemophiliac patients on April 1, 2007, who had been infected with HIV by blood products in the 1980s. Data that were used included HIV RNA levels, CD4 cell count, and administered antiretroviral drugs at the first quarter (from 1 April to 31 June) of the 2007 and 2014 fiscal years.

The changes in antiretroviral regimen use between 2007 and 2014 were examined. The antiretroviral regimen was classified into five categories as follows: no regimen; regimens including two nucleoside reverse transcriptase inhibitors and one or two protease inhibitors (two NRTIs + PI[s]); regimens including two NRTIs and one non-nucleoside reverse transcriptase inhibitor (two NRTIs + NNRTI); regimens including two NRTIs and one INSTI (two NRTIs + INSTI); and other regimens. The differences between HIV RNA levels in 2007 and 2014 and those between CD4 cell counts in 2007 and 2014 for the total participants and those with use of two NRTIs + INSTI were examined. Proportions of HIV RNA <50 copies/mL were calculated and the differences were tested using the McNemar test. The median, and 25th and 75th percentiles of CD4 cell counts were calculated and the differences were tested using the sign test. Progression to AIDS or death was assumed as the highest level in HIV RNA and as the lowest level in CD4 cell count. All analyses were performed using the SAS statistical package, version 9.3 (SAS, Institute, Cary, NC, USA).

### Ethical Considerations

This study was approved in June 2015 by the Ethical Review Board for Clinical Studies of National Hospital Organization Osaka National Hospital, Osaka, Japan (No. 15026).

## RESULTS

The participants were 564 men aged between 23 and 71 years old (mean, 37.7 years old) at 1 April 2007. Of these, 79 progressed to AIDS or death by 31 June 2014. Table **1** shows the numbers of participants with antiretroviral regimen use in 2007 and 2014. The proportion of use of two NRTIs + INSTI dramatically increased from 0.0% to 41.0% over this time. Proportions of each use of no regimen, two NRTIs + PI(s), two NRTIs + NNRTI, and other regimens declined over this time. The numbers of participants with changes in regimen use from no regimen, two NRTIs + PI(s), two NRTIs + NNRTI, and other regimens to two NRTIs + INSTI were 50, 110, 36, and 34, respectively.

Table **2** and Fig. (**1**) show proportions of HIV RNA levels <50 copies/mL in 2007 and 2014. The proportion of HIV RNA levels <50 copies/mL for a total of 548 participants (included 79 cases of progression to AIDS or death, but not 16 cases with missing data of HIV RNA levels) significantly increased from 63.3% in 2007 to 74.5% in 2014. The proportion of participants with a change to use of two NRTIs + INSTI significantly increased from 58.3% to 90.6% over this time. The proportion of participants with changes in antiretroviral therapy use from no regimen, two NRTIs + PI(s), two NRTIs + NNRTI, and other regimens to two NRTIs + INSTI significantly increased over this time.

Table **3** and Fig. (**2**) show the median, and 25th and 75th percentiles of the CD4 cell count in 2007 and 2014. The median CD4 cell count for a total of 543 participants (included 79 cases of progression to AIDS or death, but not 21 cases with missing data of CD4 cell count) significantly increased from 388/μL to 437/μL over this time. The median CD4 cell count of participants with a change to use of two NRTIs + INSTI significantly increased from 380/μL to 438/μL over this time. The median CD4 cell count of participants with changes in antiretroviral regimen use from no regimen, two NRTIs + PI(s), two NRTIs + NNRTI, and other regimens to two NRTIs + INSTI increased. Additionally, the median CD4 cell count for those from two NRTIs + PI(s) to two NRTIs + INSTI significantly increased.

## DISCUSSION

The proportion of use of antiretroviral regimens with an INSTI in our participants dramatically increased from 0.0% to 41.0% between 2007 and 2014. The reason for not using these regimens in 2007 is that, the first INSTI (raltegravir) was approved for treatment of HIV infection in 2008 in Japan [5]. The increase in use of regimens between 2007 and 2014 was mainly caused by the recommendations for switching to antiretroviral regimens with INSTIs in the setting of viral suppression and viral failure in guidelines in the USA, Europe, and Japan [3, 5, 11]. Our participants included more than 80% of people alive on April 1, 2007 who were infected by blood products in the 1980s in Japan. Many of these participants had long-term antiretroviral treatment with several drugs [14-16]. The finding of a large increase in use of antiretroviral regimens with an INSTI in the present study showed that there was a large effect of INSTIs in clinical practice for Japanese patients with HIV on long-term antiretroviral treatment.

A large improvement in HIV RNA levels and CD4 cell count for patients with HIV and changes in use of antiretroviral regimens with INSTIs between 2007 and 2014 were observed in the present study. These results are consistent with findings by previous studies [6-10]. Our results also confirmed that switching to antiretroviral regimens with INSTIs in clinical practice would lead to favorable effects of HIV RNA levels and CD4 cell count. Our findings suggested that there was a large effect of INSTIs on clinical improvement in the whole population of patients with HIV under long-term antiretroviral treatment.

This study has some limitations and problems. We used data from the Japan Cohort Study of HIV Patients Infected through Blood Products [14, 15]. Our participants were Japanese male hemophiliac patients with HIV who were infected by blood products in the 1980s, but we did not include females. Most participants appeared to have some favorable factors for switching to antiretroviral therapy (e.g., long-term, routinely good medical care, sufficient monitoring of HIV RNA levels and CD4 cell count, and relatively complete adherence of antiretroviral therapy) [14-16]. The data that were used in this study were at only one time point in each of 2007 and 2014. Participants who changed from no treatment in 2007 to antiretroviral regimen use, including INSTIs in 2014, in our study would have had initiation of antiretroviral regimen use, including INSTIs for treatment-naive patients between 2007 and 2014. Additionally, there also would have been those who switched to antiretroviral regimens with INSTIs after initiation of an antiretroviral regimen without INSTIs between 2007 and 2014. We observed changes in antiretroviral regimen use between 2007 and 2014. Currently, antiretroviral regimens are being continually developed, research on these regimens is ongoing, and guidelines are constantly being updated [3, 5, 11]. In Japan, other INSTIs, such as elvitegravir and dolutegravir, were approved in 2013 and 2014, respectively [5]. The current antiretroviral regimens with INSTIs have a risk of viral failure because of drug-resistance, similar to other regimens [3]. Changes in antiretroviral regimen use will continue in the future [3]. We analyzed HIV RNA levels and CD4 cell count as effects of switching to an antiretroviral regimen with an INSTI. Evaluating effects of switching regimens requires observations of other variables, such as adverse events and quality of life [3, 12].

## CONCLUSION

We found a considerable effect of switching to antiretroviral regimens with INSTIs for Japanese patients with HIV who were infected by blood products. This finding suggests that this switch in clinical practice will lead to favorable effects.

## Figures and Tables

**Fig. (1):**
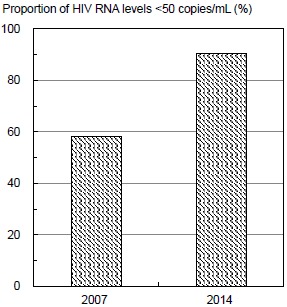
Proportion of HIV RNA levels <50 copies/mL in 2007 and 2014 for patients with HIV who used a regimen, including an integrase strand transfer inhibitor.

**Fig. (2):**
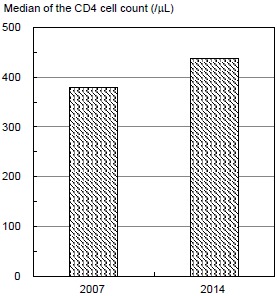
Median of the CD4 cell count in 2007 and 2014 for patients with HIV who used a regimen, including an integrase strand transfer inhibitor.

**Table 1:** Number of participants with antiretroviral regimen use in 2007 and 2014.

		**Antiretroviral regimen use in 2014**						**Progression to AIDS or** **death**	**Total (%)**	
		**No ** **regimen**	**Two NRTIs ** **+ PI(s)**	**Two NRTIs** **+ NNRTI**	**Two NRTIs ** **+ INSTI**	**Other regimens**	**Unknown**			
**Antiretroviral regimen use** **in 2007**	**No regimen**	26	13	11	50	2	0	12	114	(20.2)
	**Two NRTIs** ** + PI(s)**	1	81	14	110	13	1	33	253	(44.9)
	**Two NRTIs** ** + NNRTI**	1	5	46	36	3	2	16	109	(19.3)
	**Two NRTIs** ** + INSTI**	0	0	0	0	0	0	0	0	(0.0)
	**Other** ** regimens**	2	10	3	34	16	1	12	78	(13.8)
	**Unknown**	1	1	0	1	1	0	6	10	(1.8)
**Total**		31	110	74	231	35	4	79	564	(100.0)
**(%)**		(5.5)	(19.5)	(13.1)	(41.0)	(6.2)	(0.7)	(14.0)	(100.0)	

Two NRTIs + PI(s): regimens including two nucleoside reverse transcriptase inhibitors (NRTIs) and one or two protease inhibitors. Two NRTIs + NNRTI: regimens including two NRTIs and one non-nucleoside reverse transcriptase inhibitor. Two NRTIs + INSTI: regimens including two NRTIs and one integrase strand transfer inhibitor. Progression to AIDS or death was between 1 April 2007 and 31 June 2014.

**Table 2:** Proportion of HIV RNA levels <50 copies/mL in 2007 and 2014.

**Antiretroviral regimen use**		**No.**	**Proportion of HIV RNA levels <50 copies/mL (%)**		**P value**
**in 2007**	**in 2014**		**in 2007**	**in 2014**	
Total	Total	548	63.3	74.5	0.000
Total	Two NRTIs + INSTI	223	58.3	90.6	0.000
No regimen	Two NRTIs + INSTI	48	6.3	89.6	0.000
Two NRTIs + PI(s)	Two NRTIs + INSTI	105	76.2	87.6	0.019
Two NRTIs + NNRTI	Two NRTIs + INSTI	36	75.0	97.2	0.027
Other regimens	Two NRTIs + INSTI	34	58.8	94.1	0.002

Two NRTIs + PI(s): regimens including two nucleoside reverse transcriptase inhibitors (NRTIs) and one or two protease inhibitors. Two NRTIs + NNRTI: regimens including two NRTIs and one non-nucleoside reverse transcriptase inhibitor. Two NRTIs + INSTI: regimens including two NRTIs and one integrase strand transfer inhibitor. P value of McNemar test for differences between proportions of HIV RNA levels <50 copies/mL in 2007 and 2014.

**Table 3:** Median, and 25th and 75th percentiles of the CD4 cell count in 2007 and 2014.

**Antiretroviral ** **regimen use**		**No.**	**CD4 cell count (/μL)**						**P value**
			**in 2007**			**in 2014**			
**in 2007**	**in 2014**		**Median**	**25th** **percentile**	**75th** **percentile**	**Median**	**25th** **percentile**	**75th** **percentile**	
Total	Total	543	388	262	552	437	265	606	0.024
Total	Two NRTIs + INSTI	227	380	264	539	438	318	630	0.000
No regimen	Two NRTIs + INSTI	49	389	295	446	421	338	533	0.085
Two NRTIs + PI(s)	Two NRTIs + INSTI	109	381	259	582	460	307	642	0.007
Two NRTIs + NNRTI	Two NRTIs + INSTI	35	380	222	673	514	335	823	0.090
Other regimens	Two NRTIs + INSTI	34	369	268	524	408	207	629	0.121

Two NRTIs + PI(s): regimens including two nucleoside reverse transcriptase inhibitors (NRTIs) and one or two protease inhibitors. Two NRTIs + NNRTI: regimens including two NRTIs and one non-nucleoside reverse transcriptase inhibitor. Two NRTIs + INSTI: regimens including two NRTIs and one integrase strand transfer inhibitor. P value of the sign test for differences between CD4 cell counts in 2007 and 2014.
